# Family Burden of ICU Survivors and Correlations with Patient Quality of Life and Psychometric Scores – A Pilot Study

**DOI:** 10.2478/jccm-2022-0027

**Published:** 2022-11-12

**Authors:** Vassiliki Mantziou, Charikleia S. Vrettou, Alice G. Vassiliou, Stylianos E. Orfanos, Anastasia Kotanidou, Ioanna Dimopoulou

**Affiliations:** 1 Department of Critical Care Medicine and Pulmonary Services, School of Medicine, National and Kapodistrian University of Athens (NKUA), “Evangelismos” Hospital, Athens, Greece

**Keywords:** quality of life, critical illness, post intensive care syndrome, family burden

## Abstract

**Introduction:**

Post intensive care syndrome (PICS) affects an increasing number of critical illness survivors and their families, with serious physical and psychological sequelae. Since little is known about the burden of critical illness on ICU survivor families, we conducted a prospective observational study aiming to assess this, and investigate correlations of the patients’ psychometric and health-related quality of life (HRQOL) scores with family burden.

**Materials and Methods:**

Twenty-nine patients were evaluated in the presence of a family member. Participants were assessed with the use of validated scales for anxiety, depression, post-traumatic stress disorder, cognitive decline, and the family burden scale (FBS).

**Results:**

High burden was present in 27.6% of family members. Statistically significant correlations were observed between the FBS score and trait anxiety, depression, and the physical and psychological components of HRQOL.

**Conclusions:**

Our results suggest that family burden following critical illness is common, suggesting that its assessment should be incorporated in the evaluation of PICS-family in large observational studies.

## Introduction

Intensive care unit (ICU) mortality is decreasing nowadays, due to the scientific and technological advances in medicine. While the number of ICU survivors increases, the long-term effects of critical illness on survivors are also increasingly recognized and studied. The physical and psychological consequences of critical illness are described with the term “Post- intensive care syndrome” [1, 2]. The presence of similar symptoms, particularly those affecting the mental and social sphere, have been also recognized in the families and caregivers of such patients, and the term PICS-Family (PICS-F) is used in the medical literature [3]. Different instruments have been used in various studies to assess the presence of such symptoms, at different time points following ICU discharge [4]; the optimum tool for the assessment of the burden of critical illness on the family domain remains to be identified. With the term burden, we refer to severe psychosocial and emotional problems, stressful situations or substantial life changes, which may affect the mental health balance of family members and caregivers of the individuals with illness [5, 6]. Despite the broad agreement of its importance, data on family and caregiver burden of ICU survivors remain scarce [7].

In the present study we used the family burden scale (FBS), to assess the presence of burden in the families of ICU survivors. FBS is an inclusive measurement tool for the assessment of the burden experienced by the caregivers of mentally ill patients, and in particular, of the caregivers of psychotic patients, and is simple in its administration [5]. We aimed at evaluating the presence of family burden, and possible correlations with the psychometric characteristics of the patients, and more specifically with anxiety and depression symptoms, post-traumatic stress disorder (PTSD), and with their perceived health related quality of life.

## Materials and Methods

### Study population

The interviews took place between May 1^st^ 2020 and October 31^st^ 2020, with patients who had been discharged 1 year ago from the ICU (ICU admission period 1/5/2019-31/10/2019). Eligibility criteria included admission in the ICU 1 year ago, patient age between 18 and 68 years, patient requiring endotracheal intubation and mechanical ventilation beyond three days, survival of the patient at the time of the interview, and the presence of a family member or caregiver at the interview. Exclusion criteria were the inability of the patient or the relative to be present at the interview due to long distance residency, the inability of the patient to communicate, or if the patient was still in a rehabilitation centre. Eligible patients were assessed for participation by telephone, and if they and their relatives agreed, an appointment for psychometric assessment was programmed. The patients’ demographics, the ICU admission cause, the length of ICU stay, previous medical history, and comorbidities were retrieved from the Hospital’s electronic medical records. During the interview, information on family and educational status, and occupation information were collected. During the interviews, the patients and relatives were additionally asked to complete relevant questionnaires. The interviews were conducted by a trained psychologist. The study was performed according to the Declaration of Helsinki guidelines and was approved by the Hospital’s Ethics Committee (protocol number 220, date of approval 21/6/2018). Informed written consent was obtained from all study participants.

### Questionnaires

The following questionnaires were administered to the patients during the interviews: A) The World Health Organization Quality of Life (WHOQOL-Bref) Questionnaire for the Assessment of the Quality of Life. The WHOQOL-Bref questionnaire has been developed for cross-cultural comparisons of quality of life, designed to assess the health related quality of life of individuals over the preceding two weeks. It has been validated for the Greek population [8]. It is comprised of 26 questions on the individual’s perception of health and wellbeing. The WHOQOL-Bref questionnaire covers four domains, including physical and psychological health, social relationships, and environmental quality of life. The responses are on a 1–5 Likert scale, which are converted to a 1–100 score according to an equation provided in the instructions. B) The State-Trait Anxiety Inventory (STAI), a 40-item questionnaire that assesses two types of anxiety; state (a temporary assessment of the anxiety levels a person is currently experiencing), and trait (a predisposition to anxiety). STAI has been validated for the Greek population [9]. A cut-off score of 40 in both the state and trait subscales define probable anxiety [10]. C) The Center for Epidemiologic Studies Depression (CES-D) scale was developed specifically for research use in the general population. Scores range from 0 to 60, with higher scores designating greater distress [11]. It has been validated for the Greek population [12]. D) The Impact of Event Scale-Revised (IES-R), designed as a measurement of post-traumatic stress disorder (PTSD) symptoms. It is a brief, self-report questionnaire. The scale has been validated for the Greek population [13]. The total score ranges from 0–88. A score above 33 indicates a possible PTSD diagnosis. Finally, E) the Family Burden Scale (FBS), which was administered to the relatives of ICU survivors [5]. This scale is comprised of 23 questions, assessing the financial burden, the effect on daily activities and social life, the presence of aggressive behavior, and the effects on family members’ health and behavior. FBS is divided in four dimensions; Factor A, measuring the impact on daily activities/ social life, comprised of eight items, and defined in terms of burden experienced from disruption of daily/ social activities. Factor B (four items), measures aggressiveness in terms of episodes of aggression, violence and serious damages at home. Factor C (six items), measures the impact on health, and indicates psychopathological signs and symptoms, as reported by the family or caregiver. Factor D (five items), is a measurement of economic burden defined as financial problems arising from the patient’s illness. The items comprising factors A, B, and D measure objective burden, while the items of factor C refer to subjective measures. The ratings are made on a three-point scale, selecting between “often” (0 points), “sometimes” (1 point), and “never” (2 points). A total score is calculated ranging from 0-46, with higher scores signifying increasing burden. The best cut-off point for a pathologically high family burden is described to be 24, in the overall scale.

### Statistics

Data are given as N (%), mean ± standard deviation (SD), or median (interquartile range, IQR), as appropriate. Comparisons between patients and healthy controls were performed with the non-parametric Mann–Whitney test. Correlations were performed by Spearman’s correlation coefficient. All analyses were performed using the IBM SPSS statistics 26 software (SPSS, Chicago, IL, USA; version 26.0). p< 0.05 was considered significant.

## Results

**Fig. 1:**
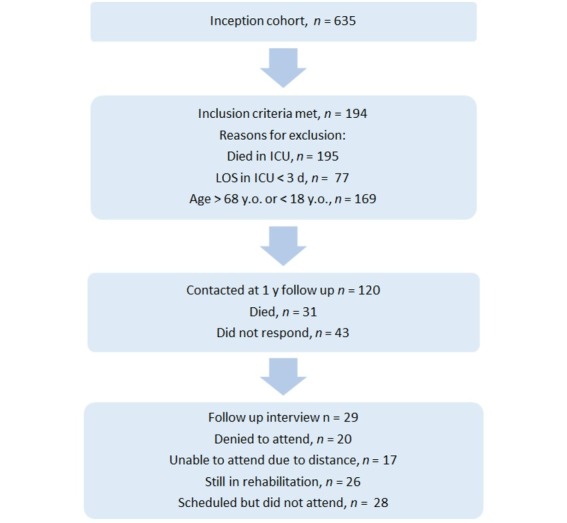
Study enrolment flowchart. ICU: Intensive Care unit; LOS; Length of Stay

**Fig. 2:**
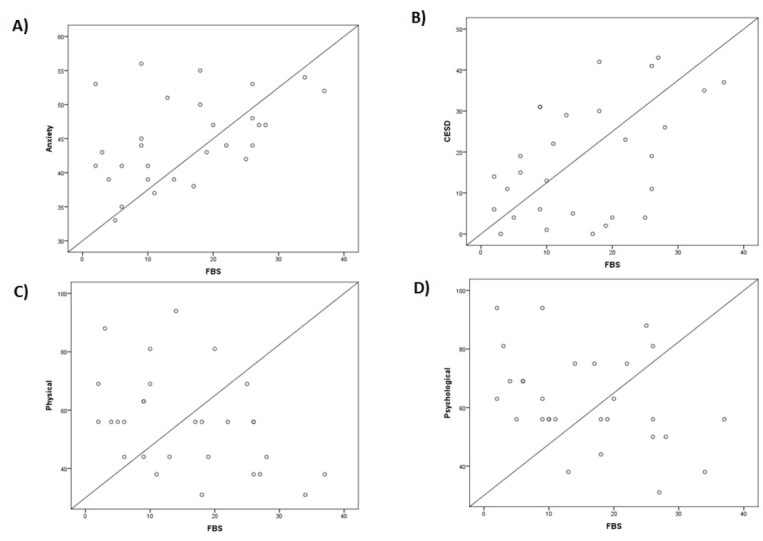
Spearman’s correlations of FBS scores. FBS: Family burden scale; CES-D: The Center for Epidemiologic Studies– Depression scale; WHOQOL: WHO Quality of life.

Six hundred and thirty-five patients were evaluated for participation. The study enrolment flow chart is given in Figure 1. Finally, 29 eligible patients were interviewed together with a family member and were included in the study. Of these patients, 16 (55.2%) were accompanied by their partners, 2 (6.8%) by their children, and 11 (38%) by their parents. The majority of patients were women, with a surgical diagnosis on admission, had higher education, were married, employed full time at the time of the interview, and would rate their health status as very good or excellent (Table 1). As can be seen in Table 1, the patients scored exceptionally high in the STAI score for both state and trait anxiety, with median values higher than the cut-off point. Twenty-two patients (75.8%) scored above the cut-off point in the trait STAI scale, 10 patients (34.4%) scored above the cut-off point in the CES-D, and 13 patients (44.8%) scored above the cut-off point in the IES-R scale. The results for the different psychometric tests are presented in Table 1. The median value for the score of the family burden scale (FBS) was 14 (7.5-25.5). In 8 cases (27.6%) the FBS score was above the suggested cut-off point of 23, implying the presence of severe family burden. The highest scores were observed in the domains of social life, 6 (2-10) and health, 6 (2-9.75) followed by the economic domain. We found statistically significant positive correlations between the FBS score and trait anxiety (r_s_= 0.438, p= 0.017) (Figure 2a), depression as measured by the CES-D (r_s_= 0.410, p= 0.027) (Figure 2b), and negative correlations with the physical and psychological domains of the WHOQOL-Bref questionnaire (r_s_= -0.438, p= 0.017, for both) (Figure 2c & d). The families of unemployed and retired patients, as well as of patients of higher educational status, scored higher in the FBS [23 (14-27) vs 9 (4.25-17), p= 0.003 and 25.5 (18-29.5) vs 9.5 (5.75-14.75), p = 0.001; respectively). There were no statistically significant differences in the FBS scores between parents, children or spouses of patients (p= 0.47).

**Table 1:** Clinical Characteristics and Psychological evaluation of the patients

Variables	Results
Age ^a^	46.62 ± 14.7

Sex (male) ^b^	19 (65.5%)

ICU diagnosis of admission ^b^	
Medical	5 (17.2%)
Surgical	14 (48.3%)
Trauma	10 (34.5%)

Comorbidities Present ^b^	21 (72.4%)

ICU length of stay (days)	21.59 ± 15.8

Education	
<High school	5 (17.2%)
High school	4 (13.8%)
>High school	20 (68.9%)

Family status ^b^	
Single	12 (41.4%)
Married	15 (51.8%)
Estranged	1 (3.4%)
Divorced	1 (3.4%)

Living with others (Yes) ^b^	29 (100%)

Employment ^b^	
Full time employed	17 (58.6%)
Student	3 (10.3%)
Housekeeping	2 (6.9%)
Retired	4 (13.8%)
Unemployed	3 (10.3%)

Self-rated health Status	
Excellent	4 (13.8%)
Very good	16 (55.2%)
Good	6 (20.6%)
Fair	2 (6.9%)
Poor	1 (3.5%)

The State-Trait Anxiety Inventory (STAI) ^c^	
State-STAI score	50 (45-58)
Trait-STAI score	44 (40-50)

The Center for Epidemiologic Studies-Depression (CES-D) scale	15 (4.5-30.5)

The Impact of Event Scale-Revised (IES-R) score	27.5(5.75-46.25)

The Mini-Mental State Exam (MMSE) score	28( 24-29)

World Health Organization Quality of Life (WHOQOL)-Bref ^d^	
Physical domain score	56 (44-66)
Psychological domain score	56 (56-75)
Social domain score	56 (50-78)
Environmental domain score	63 (53-72)

ICU, Intensive Care Unit; ^a^ Mean ± Standard Deviation (SD); ^b^ Absolute number (%); ^c^ Median (Interquartile Range); ^d^ At the time of the interview.

## Discussion

Our results suggest that a significant proportion of the families/ caregivers of ICU survivors, experience severe family burden, as measured by the FBS scale, even though the majority of patients in our study population had a god recovery post-ICU discharge, and were able to return to work. Higher FBS scores were related to the presence of depressive and anxiety symptoms in the patients, and the physical and psychological components of the quality-of-life assessment following ICU discharge.

Previous studies that have attempted to estimate the burden of critical illness in the psychological and social life of the family members, have reported significantly higher caregiver burden in patients with an unfavourable outcome [7]. We found a positive correlation between the FBS score and the physical and psychological components of the WHOQOL-Bref; however the self-reported health outcomes of the patients in our cohort were very good or good. The presence of psychological and physical issues may to an extent affect each other in complex ways. Psychological symptoms affect the capacity for rehabilitation, work performance, ability to work, while the presence of physical symptoms may significantly undermine psychology and morale of patients and their family members as well [14]. We did not observe a significant correlation between the FBS score and the social and environmental components of the WHOQOL-Bref. This could be due to the small sample size of our study. Nevertheless, the results of our study confirmed the importance of physical and psychological symptomatology over the conceived burden of illness [15]. While the finding of higher FBS scores in the families of retired and unemployed patients was expected due to the higher financial burden they are faced with, the significantly higher scores in patients with higher education, warrants further investigation. One likely explanation is that higher standards and expectations are raised by these families, and these in turn contribute to increased anxiety and family burden.

Significant correlations were also observed between the FBS score and the presence of anxiety, and in particular of trait rather than state anxiety as measured by the STAI scale, and furthermore, between the presence of depressive symptoms measured by the CES-D scale. Similar findings have been reported by other authors, but what is unique in our sample is the impressively high percentage of anxiety. We attribute this to the fact that all interviews were performed during the COVID-19 pandemic, when the levels of anxiety were high, even in the general population. Those previously affected by critical illness, had, therefore, additional reasons to worry about their health [16]. The high percentage of anxiety in our cohort may have affected the FBS scores of their family members significantly. These results highlight the significance of addressing the psychological symptoms of ICU survivors in an effective and timely manner, in order to improve their quality of life, and to diminish the burden on families [17].

Previous studies have shown that the symptoms of PICS-F may be present for months and even up to 8 years after the discharge of the patient from the ICU [3]. The frequency and severity of symptoms in the caregiver population varies greatly amongst different studies, and this has been attributed to the different methodologies, and more specifically to the different instruments used to assess PICS-F, at different time points following ICU discharge [4, 18]. The most frequently reported mental health symptoms of the family members are anxiety, depression, PTSD and/or complicated grief [19, 20, 21, 22, 23, 24]. It has been reported that symptoms of anxiety existed in half of the family members in a 6-month period [19, 20, 22, 24], while depression symptoms were also typical, however declined over time [21, 23]. These studies, however did not address family burden per se, but performed a rather general assessment of the presence of psychological symptomatology in the caregivers. Even though this approach seems reasonable, it neglects important aspects of burden, such as the economic burden and the presence of a violent behavior at home [5].

Our study has several limitations. The main limitation was that the response rate was low (4.6%), resulting in a small sample size, allowing only an initial assessment of the frequency and severity of family burden in an ICU population, and limiting the generalizability of our findings. Further prospective studies need, in our opinion, to incorporate the assessment of family burden in their design. Secondly, a more extended examination of the caregivers with the addition of more psychometric tests would enable the better evaluation of the FBS score and its performance in the specific population of the ICU survivors’ families. Finally, only evaluating the cases who presented for assessment, poses the risk of selection bias.

## Conclusions

This study provides preliminary evidence supporting the use of burden scales in the assessment of PICS-F, and this addition could impact the planning and installation of appropriate alleviating strategies.
